# Trait-related decision making impairment in obsessive-compulsive disorder: evidence from decision making under ambiguity but not decision making under risk

**DOI:** 10.1038/srep17312

**Published:** 2015-11-25

**Authors:** Long Zhang, Yi Dong, Yifu Ji, Rui Tao, Xuequan Chen, Jianguo Ye, Lei Zhang, Fengqiong Yu, Chunyan Zhu, Kai Wang

**Affiliations:** 1 Department of Neurology, The First Affiliated Hospital of Anhui Medical University, Hefei, China; 2 Laboratory of Neuropsychology, Anhui Medical University, Hefei, China; 3 Mental Health Center of Anhui Province, Hefei, China; 4 Psychological Consultation Center of Anhui Medical University, Hefei, China

## Abstract

This study aimed to investigate whether deficits in decision making were potential endophenotype markers for OCD considering different phases of the disease. Fifty-seven non-medicated OCD patients (nmOCD), 77 medicated OCD patients (mOCD), 48 remitted patients with OCD (rOCD) and 115 healthy controls were assessed with the Iowa Gambling Task (IGT), which measured decision making under ambiguity, and the Game of Dice Task (GDT), which measured decision making under risk. While the three patients groups showed impaired performance on the IGT compared with healthy controls, all patients showed intact performance on the GDT. Furthermore, the rOCD patients showed a preference for deck B, indicating that they showed more sensitivity to the frequency of loss than to the magnitude of loss, whereas the mOCD patients showed a preference for deck A, indicating that they had more sensitivity to the magnitude of loss than to the frequency of loss. These data suggested that OCD patients had trait-related impairments in decision making under ambiguity but not under risk, and that dissociation of decision making under ambiguity and under risk is an appropriate potential neurocognitive endophenotype for OCD. The subtle but meaningful differences in decision making performance between the OCD groups require further study.

Obsessive-compulsive disorder (OCD) is a phenotypically heterogeneous neuropsychiatric disorder. The pathophysiology of OCD is not well understood, and classical genetic linkage and association studies have not yet provided consistent results to identify the contributory genes involved in OCD^1^. It has been argued that the underlying neurobiology and genetic mechanisms of complex psychiatric disorders, including OCD, may be better understood by identifying potential endophenotypes^2^,^3^. Endophenotypes are intermediate phenotypes that are not obvious or external but, rather, are microscopic and internal^3^. Specifically, endophenotypes are described as heritable quantitative traits believed to be internal phenotypes mediating on a path between disease phenotypes and the biological processes underlying them^2^. Endophenotypes are advantageous in assisting with the genetic dissection of complex psychiatric disorders and provide a special approach for searching for susceptibility genes, as they represent deconstruction of the clinical phenotype into measurable disease-associated traits hypothetically more proximal to genetic effects^3^,^4^.

Neuropsychological impairments are potential endophenotype candidates in various psychiatric diseases^5^. In the context of OCD, studies have largely examined neuropsychological function in patients with medication during the symptomatic phase^5^,^6^,^7^,^8^. It is now accepted that OCD is associated with substantial impairments in neurocognitive function. Decision making is an important domain of neurocognitive function. However, individuals with OCD frequently experience serious impairment in everyday decision making; that is, making decisions appears to be dysfunctional in clinical OCD settings in the context of obsessive doubting and uncertainty^9^. In addition, there is substantial variability in the decision making strategies displayed by different individuals, and OCD is thought to result from decision making abilities being impaired^10^. Some researchers even regard decision making impairment to be the underlying cause of obsessive and compulsive symptoms^9^,^11^. Therefore, neuropsychological research into decision making in OCD patients has received considerable attention, with many of these studies highlighting impaired decision making as a potential marker of this disorder^12^,^13^,^14^,^15^.

Decision making refers to the process of selecting a particular option from a set of alternatives expected to produce different outcomes^16^. To date, from a neuroscientific perspective there are at least two types of decision making that differ mainly in the degree of uncertainty, and how much useful information about consequences and their probabilities is provided to the decision maker^17^. In some situations, outcomes and probabilities are implicit, and the decision makers have to initially find effective information and determine the qualities of the options independently by processing feedback from previous choices. This type of decision making is often termed decision making under ambiguity and is usually measured with the Iowa Gambling Task (IGT)^18^,^19^. In this task participants, who are presented with a number of decks and series of cards from which they must make choices, are unaware of the quantity of cards they need to choose or which card decks are disadvantageous (i.e., coupling large gains with even larger losses and leading to a negative overall balance in the long term) or advantageous (i.e., coupling small gains with even smaller losses and leading to a positive overall balance in the long term). In contrast with decision making under ambiguity, explicit information about the potential consequences of various choices and their probabilities is provided in some decision situations. This type of decision making is referred to as decision making under risk and can be measured with the Game of Dice Task (GDT)^20^. The GDT requires subjects to decide between options that are explicitly related to a specific amount of gain/loss. Winning probabilities are obvious and stable from the beginning of the task. Some options, related to high potential gains/losses but low winning probabilities, are risky; other options, related to lower potential gains/losses but higher winning probabilities, are non-risky. Thus, subjects are able to estimate the risk related to each option and apply strategies to maximize profit.

One study investigating decision making under ambiguity measured by the IGT and decision making under risk measured by the GDT found that although the performance of OCD patients was lower than comparison subjects on the IGT, they performed equally on the GDT^14^. Furthermore, another study found that unaffected siblings of OCD patients showed similar performance with OCD probands; that is, both OCD probands and their unaffected siblings had deficits in the IGT compared to control subjects, whereas they showed intact performance in the GDT^15^.

As we know, OCD is related to impairments in neuropsychological and neuroimaging studies, but results are inconsistent across studies. These inconsistencies may be attributable to methodological issues, such as variation in the medication status of patients, as most studies have been carried out in drug-treated patients^21^. Many studies have suggested that OCD patients have stable specific impairments of neuropsychological function, some of which may improve after treatment^22^,^23^. In one meta-analysis, Kuelz *et al.*^24^ found that medicated OCD patients had worse performance on information processing tests compared with unmedicated OCD patients, thus emphasizing the importance of carrying out studies on unmedicated OCD patients.

In the context of OCD, there is very little research on patients in the asymptomatic/remitted phase, with studies largely examining neuropsychological function in symptomatic patients. Nevertheless, commonly accepted criteria for an influenced endophenotype include trait identification in an objective and quantitative manner in patients before onset of the disorder and/or during periods of remission^13^. Meanwhile, recent studies have highlighted the link between simple tests of neuropsychological function and different phases of OCD. One study found that OCD patients had significantly higher attention bias for negative OCD stimuli, but the emotional interferences were present only in symptomatic patients and not patients in remission^25^. Conversely, other studies have shown that OCD patients in the recovered phase have significant deficits in certain executive functions and nonverbal memory, with these findings demonstrating that specific neuropsychological deficits are state independent and remain unchanged in the remitted state, possibly supporting the utility of these specific neuropsychological deficits as candidate endophenotype markers for OCD^4^,^26^.

Although decision making impairments have been reported in OCD patients^15^,^27^, the nature and extent of decision making dysfunction across phases of the disorder remain unclear. The aim of this study was to investigate if patients with OCD in different phases have decision making problems. Therefore, we assessed decision making under ambiguity measured by the IGT and decision making under risk measured by the GDT in OCD patients in three phases: non-medicated symptomatic OCD patients, medicated symptomatic OCD patients and medicated patients in remission. In line with previous findings^14^,^15^, we predicted that OCD patients in the three phases would have impairments for the IGT in comparison with matched healthy controls, but they would show intact performance for the GDT.

## Methods

### Participants

The study sample included 182 OCD patients (87 women and 95 men; age range: 18–49 years) and 115 healthy controls (55 women and 60 men; age range: 18–50 years). The patients were recruited from outpatients of the Mental Health Center of Anhui Province in Hefei, China and the Psychological Consultation Center of Anhui Medical University. Diagnostic assessment of the OCD patients was initially performed by two experienced psychiatrists and was confirmed using the Structural Clinical Interview for DSM-IV-TR^28^. Obsessive-compulsive symptom severity was assessed by the Yale–Brown Obsessive Compulsive Scale (Y-BOCS)^29^. The 14-item Hamilton Anxiety Rating Scale (HARS)^30^ and the 17-item Hamilton Depression Rating Scale (HDRS)^31^ were used to assess the current anxiety and depressive symptoms of the OCD patients. OCD subjects were excluded if they: 1) met any other DSM-IV-TR axis I diagnosis, including lifetime history of depression; 2) had a HDRS score >8; or 3) had a HARS score >13^4^. All participants gave written informed consent. The study was executed in agreement with the Declaration of Helsinki and approved by the Ethics Committee of Anhui Medical University. None of the subjects had received cognitive behavior therapy previously.

The patients with OCD were divided into three groups: patients with non-medicated OCD (nmOCD), patients with medicated OCD (mOCD) and patients with medicated OCD in remission (rOCD).

OCD patients were included in the nmOCD group if they: 1) met the DSM-IV-TR diagnostic criteria for OCD; 2) had never been treated with any psychiatric medication; 3) had a Y-BOCS total severity score ≥16; and 4) had at least 6 years of school education. The nmOCD group totaled 57 patients comprising of 30 women and 27 men (age range: 18–48 years).

Patients were included in the mOCD group: 1) met the DSM-IV-TR diagnostic criteria for OCD; 2) had been on treatment with serotonin reuptake inhibitors (SRIs) at an adequate dose for at least 12 consecutive weeks^32^; 3) had a Y-BOCS total severity score ≥16; and 4) had at least 6 years of school education. The mOCD group totaled 77, consisting of 42 women and 35 men (age range: 18–47 years). All 77 patients were on treatment with SRIs (fluoxetine, 22; sertraline, 18; paroxetine, 16; citalopram, 6; fluvoxamine, 6; clomipramine, 5; and escitalopram, 4). Of the 77 patients, 14 were receiving benzodiazepines (clonazepam, 9; and estazolam, 5) and 21 were on antipsychotic augmentation (risperidone, 11; olanzapine, 4; quetiapine, 4; and aripiprazole 2).

OCD patients were included in the rOCD group: 1) had met the DSM-IV-TR diagnostic criteria for OCD at baseline, but were currently in a remitted state; and 2) had at least 6 years of school education. A patient was considered to be in remission if he/she had a Y-BOCS total severity score <16 and did not fulfil DSM-IV-TR criteria for OCD. In addition, the patient had to acknowledge that, for at least 8 consecutive weeks, symptoms occurred for less than 1 hour per day and caused no more than mild anxiety/distress or interference in functioning^33^,^34^. The rOCD group totaled 48 patients: 23 women and 25 men (age range: 18–49 years). All 48 patients were on treatment with SRIs (fluoxetine, 13; sertraline, 10; paroxetine, 8; citalopram, 5; fluvoxamine, 6; clomipramine, 3; and escitalopram, 3). Of the 48 patients, 10 were receiving benzodiazepines (clonazepam, 8; and estazolam, 2). Twelve of them were on antipsychotic augmentation (risperidone, 6; olanzapine, 3; quetiapine, 2; and aripiprazole 1). Importantly, there was no significant difference in medication type and duration of treatment between the mOCD and rOCD groups.

A total of 115 participants without a known family history of OCD were recruited as healthy controls (HC) by advertisements, leaflets or word of mouth from college students and the local community. They were matched for age, gender and education with participating patients. The exclusion criteria were current or past diagnosis of any psychiatric disorder, neurological illness, head injury, drug or alcohol abuse, gambling addiction, having serious medical illness, or the consumption of drugs known to affect cognition.

### Neuropsychological background tests

#### Digit Span test

Verbal short-term memory and verbal working memory were tested by the Digit Span Test (DST)^35^. In DST forward the participants are told to repeat the same sequence as had been read by the examiner, whereas in DST backward the participants are told to repeat the sequence in reverse order as had been read by the examiner.

#### Trail Making Test

All participants had completed the Trail Making Test (TMT)^36^: Test A and Test B. For Test A, participants were asked to connect 25 encircled numbers, which were distributed on a piece of paper, as accurately and quickly as possible in ascending order. For Test B, participants were asked to connect numbers and letters alternately (e.g., 1, A, 2, B, 3, C, etc.). If a mistake was made, the participant could return to the “circle” where the mistake originated and continue. Test A measures mental tracking and motor speed, and Test B captures selective attention and cognitive flexibility. The amount of time required to complete each test represents the score on each test.

#### Wisconsin Card Sorting Task

Participants in the three groups had also completed the Wisconsin Card Sorting Task (WCST)^37^, which measures executive function. The computerized version of WCST was used. The test consists of four different types of stimulus cards (triangle, star, cross and circle). Participants are given a set of target cards and requested to detect sorting principles (form, color and number) and to match each target card with one of the four stimulus cards. However, the sorting pattern changes after 10 sequential correct responses and participants must switch to a new sorting pattern based on the feedback (correct or incorrect). After 128 trials or when participants achieved nine reversals, the task ends. The total sum of wrong responses, the total sum of perseverative responses, the total sum of perseverative errors are calculated for analyses.

### Decision making under ambiguity

The computerized version of the IGT was used to measure decision making under ambiguity^18^,^19^. In this task, subjects are instructed to choose one card from four decks of cards (A, B, C and D). After each card selection, they win or lose a specified amount of money. On the IGT, decks A and B yield an average gain of €100 per selection, and decks C and D yield an average gain of €50 per selection. Subjects also encounter losses. 10 selections from decks A or B lead to a net loss of €250, whereas ten selections from decks C or D lead to a net gain of €250. In short, A and B are disadvantageous decks, they include high immediate gains, but even higher losses, resulting in a negative outcome over the long run; decks C and D are advantageous, they produce small immediate gains, but even smaller losses, resulting in a positive outcome in the long term. Moreover, there are also other inequalities between the four decks. For instance, although decks A and B lead to long-term negative outcomes, selections from deck A are punished on 50% of trials but deck B selections are punished on 10% of trials. The immediate losses on deck A are also smaller than those in deck B. Similar differences are seen between decks C (50% losses) and D (10% losses), and the immediate losses on deck C are also smaller than those in deck D^38^.

Subjects are told that some decks are better than other decks and they can select cards from any deck. They are told to win as much money as possible with a starting capital over 100 trials. The gain or the loss after each selection, and the new monetary total are shown on the screen. No other information was given. We calculated the total netscore by subtracting the number of disadvantageous choices from the number of advantageous choices to analyze task performance. The 100 trials were divided into five equal blocks, and the netscore of each block of 20 cards was calculated to investigate whether decision making changed during the task. Furthermore, the number of cards selected in individual deck A, B, C and D were calculated to examine individual deck level preference.

### Decision making under risk

To assess decision making under risk, we used the computerized GDT^20^. In the task, subjects roll a virtual die 18 times, with the goal of maximizing their gains with a fictitious starting capital (€1000) by choosing one of four different options. Subjects guess the result of the game and choose to bet on either a single die or one die out of two, three or four dice combinations. They win some money if the chosen number or one of the chosen numbers is thrown, otherwise they lose the same amount of money. Each option is associated with defined gain/loss and different winning probabilities: 1000€ gain/loss with a winning probability of 1:6 for a single number; 500€ gain/loss with a winning probability of 2:6 for combination of two numbers; 200€ gain/loss with a winning probability of 3:6 for combination of three numbers; 100€ gain/loss with a winning probability of 4:6 for combination of four numbers. If, for instance, a participant bets on the combination “one”, “two” and “three”, and a one, two, or three is thrown, the participant wins 200€; however, if a four, five or six is thrown, 200€ are lost. The two former options, which have lower winning probabilities are grouped into risky decisions; the two latter options, which have higher winning probabilities are grouped into non-risky decisions. Additionally, the gain or the loss, the change in capital, and the number of the rest of die throws were presented on the screen after each selection.

For analysis, we calculated a netscore (the number of non-risky choices minus the number of risky choices) to analyze task performance. We also calculated how often the four different options were chosen.

### Statistical analysis

SPSS 16.0 was used to perform all of the statistical analyses. All of the variables were tested for normal distribution with the Kolmogorov–Smirnov Test separately for the four groups. There were no significant deviations from the normal distribution for the IGT netscore, the GDT netscore and the neuropsychological variables. Thus, parametric methods were used for these variables. A one-way analysis of variance (ANOVA) with group as the between-subjects factor was performed to examine the IGT netscore and the netscore in each block. A one-way ANOVA with block as the between-subjects factor was performed to examine the influence of decision process on the IGT netscore, and a one-way ANOVA with group as the between-subjects factor was performed to examine individual deck level preference. The GDT netscore and the effects of choice were analyzed using a one-way ANOVA with group as a factor.

## Results

### Demographic and clinical characteristics of the sample

The demographic characteristics of the subjects are shown in Table 1. No differences were found between the nmOCD, mOCD, rOCD and HC groups for age, years of education or sex. No differences were found between the nmOCD, mOCD and rOCD groups for age of onset and duration of OCD. The nmOCD and mOCD groups scored higher on total Y-BOCS scores, Y-BOCS obsessions scores, Y-BOCS compulsions scores, HARS scores and HDRS scores than the rOCD group (all *p*s < 0.001).

### Neuropsychological assessment

The neuropsychological tasks performance in the four groups are shown in Table 2. Significant differences between the four groups were present on the TMT B (*F*(3,293) = 2.85, *p* = 0.038). No differences were found between the nmOCD, mOCD, rOCD and HC groups for other neuropsychological variables (all *p*s > 0.05).

### Netscore on the IGT

**Figure 1:**
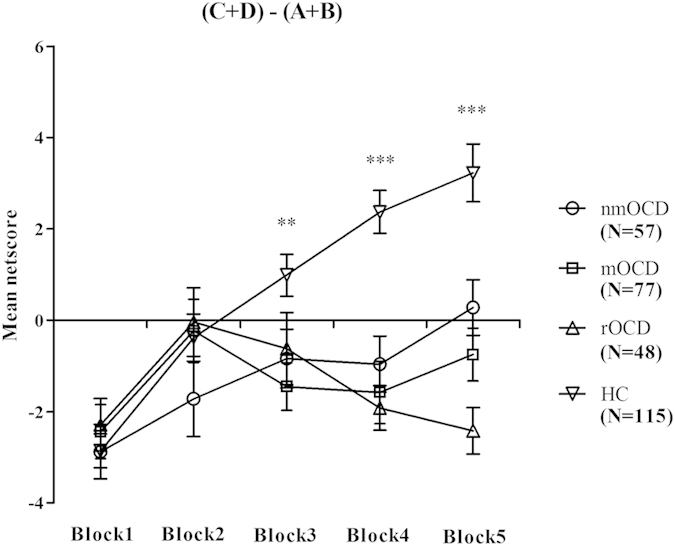
Netscore of the five blocks during the IGT. Mean netscore for each block of 20 trials for subjects with nmOCD, mOCD, rOCD and HC. **p < 0.01 and ***p < 0.001. Means ± SEMs are shown.

There were significant differences between the IGT netscores of the four groups (*F*(3,293) = 15.03, *p* < 0.001). The HC group scored higher than the nmOCD, mOCD and rOCD groups (all *p*s < 0.001), and there were no significant differences between the nmOCD, mOCD and rOCD groups (*F*(2,179) = 0.12, *p* = 0.885) (Table 3). The single comparisons of performance on the five blocks between groups indicated significant netscore differences in blocks 3, 4 and 5. See Fig. 1 and Table 3.

### Individual deck level preference on the IGT

**Figure 2:**
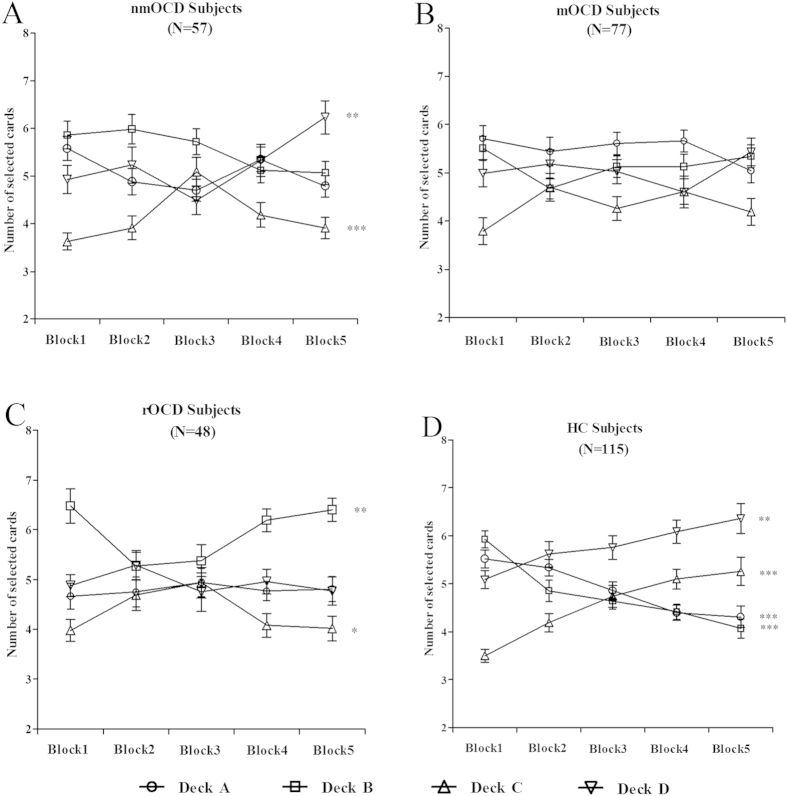
Number of cards selected in blocks during the IGT. Mean number of cards selected from individual decks A, B, C and D for subjects with nmOCD (**A**), mOCD (**B**), rOCD (**C**) and HC (**D**), graphed as a function of trial block. *p < 0.05, **p < 0.01 and ***p < 0.001. Means ± SEMs are shown.

In the IGT, the change curve of deck level indicates the changes in decision strategies. In the nmOCD group, the number of cards selected changed significantly over the course of the task in decks C (*F*(4,280) = 5.20, *p* < 0.001) and D (*F*(4,280) = 3.71, *p* = 0.006), but not in decks A and B (all *p*s > 0.05) (Fig. 2A). In the mOCD group, the number of cards selected did not change significantly over the course of the task in decks A, B, C and D (all *p*s > 0.13) (Fig. 2B). In the rOCD group, the number of cards selected changed significantly over the course of the task in decks B (*F*(4,235) = 4.10, *p* = 0.003) and C (*F*(4,235) = 2.76, *p* = 0.029), but not in decks A and D (all *p*s > 0.05) (Fig. 2C). In the HC group, the number of cards selected changed significantly over the course of the task in decks A (*F*(4,570) = 8.65, *p* < 0.001), B (*F*(4,570) = 14.25, *p* < 0.001), C (*F*(4,570) = 10.92, *p* < 0.001) and D (*F*(4,570) = 3.62, *p* = 0.006) (Fig. 2D).

**Figure 3:**
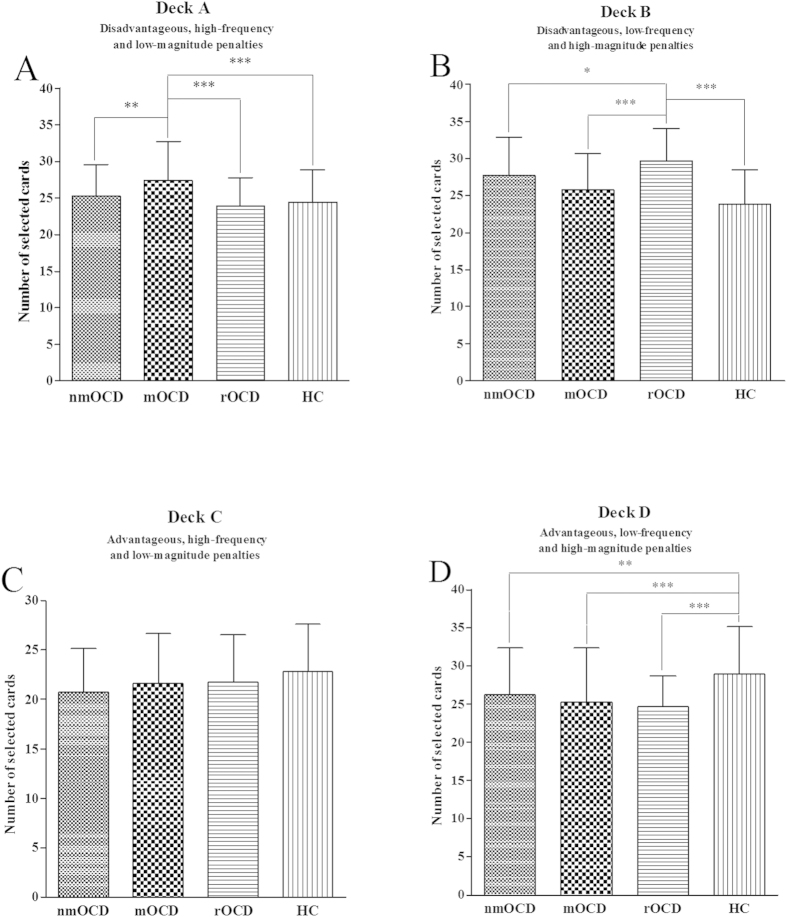
Number of cards selected for groups during the IGT. Mean number of cards selected for subjects with nmOCD, mOCD, rOCD and HC from individual decks A (**A**), B (**B**), C (**C)**, and D (**D**) over 100 picks of cards. *p < 0.05, **p < 0.01 and ***p < 0.001. Means ± SEMs are shown.

There were significant differences in deck A overall score between the four groups (*F*(3,293) = 8.73, *p* < 0.001). The mOCD group selected significantly more cards from the deck A than the nmOCD, rOCD and HC groups did (all *p*s < 0.01), with no significant differences between the nmOCD, rOCD and HC groups (all *p*s > 0.05) (Fig. 3A). There were significant differences in deck B overall score between the four groups (*F*(3,293) = 19.85, *p* < 0.001). The rOCD group selected significantly more cards from the deck B than the nmOCD, mOCD and HC groups did (all *p*s < 0.05), with no significant differences between the nmOCD, mOCD and HC groups (all *p*s > 0.05) (Fig. 3B). There were no significant differences in deck C overall score between the four groups (*F*(3,293) = 2.62, *p* > 0.05) (Fig. 3C). There were significant differences in deck D overall score between the four groups (*F*(3,293) = 8.22, *p* < 0.001). The HC group selected significantly more cards from the deck D than the nmOCD, mOCD and rOCD groups did (all *p*s < 0.01), with no significant differences between the nmOCD, mOCD and rOCD groups (all *p*s > 0.05) (Fig. 3D).

### Decision making on the GDT

In contrast to the IGT, there was no significant difference between the netscores of the four groups (*F*(3,293) = 0.05, *p* = 0.987). None of the single comparisons for the different choices reached significance between groups (all *p*s > 0.05) (Table 3).

We examined the use of negative feedback (losses) after the decision of a risky option to choose a non-risky option in the next trial; only those participants who chose a risky option and received negative feedback at least once during the GDT were included. Thus, the data of 253 subjects were analyzed. The four groups did not differ on the use of negative feedback (*F*(3,249) = 0.10, *p* = 0.96) (Table 3). The feedback use was significantly associated with the GDT netscore in the nmOCD (*r* = 0.84, *p* < 0.001), mOCD (*r* = 0.38, *p* = 0.003) and HC (*r* = 0.36, *p* < 0.001) groups, but not the rOCD group (*r* = 0.23, *p* = 0.151). We also examined the use of positive feedback (gains) after the decision of a non-risky option to choose a non-risky option again; only those participants who chose a non-risky option and received positive feedback at least once during the GDT were included. Thus, the analysis was based on the data of 289 participants. There was no significant differences between the four groups with regard to the use of positive feedback (*F*(3,285) = 0.31, *p* = 0.82) (Table 3). The use of positive feedback was also significantly associated with the GDT netscore in the nmOCD (*r* = 0.73, *p* < 0.001), mOCD (*r* = 0.50, *p* < 0.001) and HC (*r* = 0.37, *p* < 0.001) groups, but not the rOCD group (*r* = 0.16, *p* = 0.31).

## Discussion

The study yielded two main results. The primary finding was evidence of a clear dissociation of decisions under implicit versus explicit conditions in patients with OCD. While patients in the three different phases of OCD (i.e., nmOCD, mOCD and rOCD) had impairments in the IGT in comparison with matched healthy controls, all patients showed intact performance in the GDT. Furthermore, patients in the three different phases showed different individual deck level preferences in the IGT: the rOCD patients showed a preference for deck B, indicating that they showed more sensitivity to the frequency of loss than to the magnitude of loss, whereas the mOCD patients showed a preference for deck A, indicating that they had more sensitivity to the magnitude of loss than to the frequency of loss. To our best knowledge, this is the first study examining decision making under ambiguity and decision making under risk in medication free OCD subjects and recovered OCD subjects.

In the IGT, the OCD patients appeared highly motivated by the prospect of immediate gain but were insensitive to the future outcome of their behaviors. Some researchers have proposed that the ritualistic behaviors related to OCD result from a detrimental sensitivity to immediate gain without proper judgment of the long-term consequences of behaviors^39^. This pattern of strategy choice resembled that of patients with orbitofrontal cortex (OFC) damage^19^,^40^. The specificity of their choices during the IGT suggests that the preference of OCD patients for disadvantageous decks does not reflect random choice but, rather, deliberate decision making. It may be supposed that OCD patients perform in the IGT as they behave in daily life, particularly under circumstances of uncertainty and complexity, due to the presence of obsessive thinking that must be neutralized by repetitive compulsions. In this analogy, the compulsions represent the immediate rewards (relief from anxiety due to obsessions) but these rewards have consequential malfunctioning in real life^39^.

Some authors have suggested that conceptualizing OCD as a disorder of decision making allows the application of novel approaches in measuring symptom provocation and eliminating symptoms, potentially leading to new approaches for the cognitive behavioral therapy of this disorder^9^,^11^. Meanwhile, the assessment of decision making deficits as potential endophenotypes is particularly meaningful in the light of findings from previous studies that have reported deficits in decision making as being among the most consistent deficits in OCD patients^6^,^14^. In order for a cognitive measure, or any trait marker, to be considered a putative endophenotype, it must fulfill certain criteria: be associated with the disease in the population, be heritable, be independent of the clinical state, and be found in clinically unaffected first-degree relatives of patients at a higher rate than in the general population^3^.

Previous studies using the IGT in OCD patients and their relatives have suggested that deficits in decision making under ambiguity could qualify as a suitable endophenotype candidate for OCD^6^,^15^,^27^. Furthermore, a commonly accepted criterion for a potential endophenotype is trait identification in an objective and quantitative manner in patients in remission. The study of drug-naive individuals is also essential to confirm deficits in decision making as potential endophenotypes. However, in this context, most studies have examined decision making in patients on medication, during the symptomatic phase. Our study found that, irrespective of the medication status of OCD patients, deficits in decision making under ambiguity existed and remained unchanged despite symptom remittance, indicating that these deficits are trait-like in nature.

Previous studies proposed that deficits in decision making may qualify as an endophenotype candidate for OCD^6^,^27^. However, our study went a step further to simultaneously assess decisions in ambiguous and risky situations, and showed that dissociation of decision making under ambiguity and decision making under risk was a more appropriate potential neurocognitive endophenotype for OCD.

In the IGT, although decks A and B lead to long-term negative outcomes, deck A includes high-frequency and low-magnitude losses but deck B includes low-frequency and high-magnitude losses. A higher number of deck A or deck B selections depends on whether subjects focus more on the magnitude or frequency of loss^41^,^42^. Our study found that individuals with mOCD made more deck A selections, suggesting that this group focused more on the magnitude of loss. These impairments are likely related to abnormal reinforcement learning. The mOCD patients might think that they had lost the most during the high-magnitude loss condition and the least in the low-magnitude loss condition^43^. They made more use of information about loss magnitude, but simultaneously neglected information about the frequency of loss. At the same time, our study found that individuals with rOCD made more deck B selections and suggested that this group focused more on frequency of loss than magnitude of loss. Their preferential selection of the deck with large, infrequent penalties could be motivated by the attraction to the relatively high reward frequency associated with deck B^44^. The IGT performance in patients with rOCD appears to be compromised by impairment in the ability to effectively and appropriately represent the relative value of reward and loss associated with the different options and response stimuli. Rapid learning based on trial-to-trial feedback and the maintenance of this information on-line are impaired in this population^45^.

Although the OCD patients in all three groups had impairments on the IGT compared with HC, we focused on the rOCD and mOCD groups. There were no significant differences in medication type and duration of treatment between these two groups; however, they showed different individual deck level preference in the IGT, with entirely different treatment outcomes from medication. The IGT manual demonstrates that, while avoiding deck B is considered a relatively good decision, deck A should be avoided by most “neurologically intact” individuals^38^. The assessment of deck preference separately for decks A and B would allow identifying subjects who have a general impairment in decision making (preference for deck A, “pathological” decision making) versus those who are prone to risky decisions, but less impaired in decision making overall (preference for deck B)^41^.

Taken together, our results provide some interesting implications for disadvantageous decision making by OCD patients on the IGT and emphasize the importance of examining selections from individual decks separately. Studies with a prospective design and a larger sample are needed to assess whether individual deck level preference can be used as an important predictor of the effectiveness of treatment, by evaluating individual deck level preference in non-medicated OCD patients before they begin a course of medication. More significantly, it will be necessary to define whether a certain internal link exists between the level of impairment of decision making and the effectiveness of treatment.

It is sometimes unclear whether cognitive difficulties change or persist after successful treatment. Answering this question could help establish whether cognitive dysfunctions in OCD are state-dependent or trait-like^46^. Impaired decision making under ambiguity was detected in all three phases of OCD in our study. Decision making impairment is a diagnostic feature of symptomatic patients with OCD, as shown by our patients in the acute phases of OCD. Similarly, children with OCD in symptomatic phase perform poorly on the IGT^47^. The remitted patients with OCD in our study also exhibit difficulties in decision making measured by the IGT, which is in accordance with previous studies of decision making deficits assessed with this method^6^,^14^,^15^,^39^,^48^, and with the Cambridge Gamble Task^9^ in symptomatic patients. Our findings are in line with a previous study demonstrating that patients in the recovered phase of OCD have significant specific deficits in neuropsychological tests^4^. Another meaningful study reported that patients with OCD improved obviously after several weeks of intensive cognitive-behavioral psychotherapy, but these patients continued to show a reduced capacity level for implicit procedural learning^49^. All these results are further arguments for the independence of specific cognitive functions from symptom states and indicate that neuropsychological deficits are potentially candidate endophenotype markers for OCD.

To clearly understand which cognitive deficits are characteristic of OCD, we need to further compare neuropsychological performance in medication-naive, never-treated OCD patients with that of medicated patients. In our study, we reported that medication-free patients showed similarly impaired decision making under ambiguity to medicated patients, which is in accordance with some previous studies of decision making deficits in unmedicated patients assessed with the IGT^39^,^50^. Several studies have commented on the effects of atypical antipsychotics^51^, SSRIs^24^ and benzodiazepines^52^ on cognitive performance. For instance, one systematic review compared mean effect sizes of group differences in cognitive function between medicated and unmedicated OCD patients, and found that SSRIs impair speed of information processing in OCD patients^24^. However, IGT performance in medication-free OCD patients was comparable with medicated patients in our study. Our findings are also consistent with a previous study showing that SSRI-medicated patients with OCD are able to perform cognitive functioning tests at a comparable level with SSRI-free patients^52^,^53^. A similar study found that OCD patients show persistent cognitive deficits before and after treatment with fluoxetine, and suggested that cognitive impairments in OCD are not secondary to symptoms and therefore form a trait feature of this disorder^54^. The use of these medications may not affect decision making performance in OCD and these results have positive implications for OCD patients who respond to medication.

Previous studies have suggested that unimpaired IGT performance, in the sense of preferentially selecting the advantageous options, depends on intact functioning of the ventromedial prefrontal cortex (vmPFC)/OFC^19^,^40^. However, neuropsychological and neuroimaging studies have found that the dorsolateral prefrontal cortex (dlPFC) plays a major role when performing the GDT^55^,^56^. Many functional imaging and morphometric magnetic resonance imaging studies of OCD have supported the notion that abnormalities in key gray matter regions, such as the OFC, anterior cingulate cortex and striatum, play important roles in its pathophysiology^57^,^58^. Furthermore, neuroimaging studies have identified abnormally reduced activation of the lateral OFC in OCD patients and their unaffected first-degree relatives during reversal learning^59^. For the dlPFC, studies on the potential involvement of this region in the pathophysiology of OCD are inconsistent. Although some research has shown abnormalities in the dlPFC activity of OCD patients^60^, other studies have not yielded similar results^61^,^62^. Furthermore, the performance of our study subjects on the WCST was intact, and the WCST is associated with executive functioning and is primarily dependent on dlPFC functioning^63^. According to these previous findings and our results, we speculate that patients with OCD may show intact function of the dlPFC, further emphasizing that the deficits exhibited in OCD potentially occur as a result of dysfunction of the OFC.

Initially, performance on the IGT is frequently proposed as being heavily dependent on emotional feedback processing, with relatively less dependence on other executive functions. The GDT draws more primarily on specific executive function processes such as set-shifting, cognitive flexibility and categorization, as measured by the WCST^17^. Our results showed OCD patients’ poor performance on emotional decision making, contrasting with other findings of intact cognitive decision making in this patient group, which may suggest a dissociation of emotional decision making from cognitive decision making in OCD^64^.

However, follow-up studies have increasingly found that the IGT and GDT share similar emotional and cognitive processes, those of feedback processing and executive functions. On the one hand, as the IGT progresses, subjects learn the outcomes associated with different decks. At some point, the IGT becomes more explicit, and the mechanisms underlying this task are similar to those in the GDT^14^. On the other hand, performing the GDT successfully also involves an optimal use of feedback processing. But the two tasks differ in the extent to which they rely on these processes. While using feedback is more important than executive functions for determining the rules in the IGT, executive functions seem to be more important for comprehending the explicit rules and forming and utilizing some appropriate strategies, in the GDT^65^. Moreover, the shift from implicit to explicit knowledge for IGT contingencies occurred in the healthy controls, but not in the OCD patients^14^.

Limitations in our study should be acknowledged. First, it has been suggested that performing the IGT successfully depends on emotional processing^50^. Whether IGT performance in our study is also regulated by emotions should be assessed in further studies measuring the emotional reactivity of participants during the task (through skin conductance response, heart rate or pupil dilation). Second, patients with OCD were not classified into subtypes. Third, the nmOCD and rOCD groups had a relatively small number of subjects. Fourth, the study was limited in its interpretation of the potential neural mechanisms of impairments in decision making.

In summary, our study of OCD patients in three different phases of illness suggests that dissociation of decision making under ambiguity and decision making under risk are potential endophenotype markers for OCD. Further work is required to confirm our findings by coupling imaging, genomics and electrophysiological strategies, examining whether our findings are related to OCD symptom dimensions. Additionally, future work to investigate differences between patients with early- and late-onset OCD is required. Most importantly, the observation of trait-related impairment in OCD, using the IGT, is of major clinical interest; whether it represents a future therapeutic target needs further confirmation.

## Additional Information

**How to cite this article**: Zhang, L. *et al.* Trait-related decision making impairment in obsessive-compulsive disorder: evidence from decision making under ambiguity but not decision making under risk. *Sci. Rep.* **5**, 17312; doi: 10.1038/srep17312 (2015).

## Figures and Tables

**Table 1:** Demographic Characteristics of the Sample [M(S.D.)].

	nmOCD (n = 57)	mOCD (n = 77)	rOCD (n = 48)	HC (n = 115)	*F*	*P*
Age (years)	28.07 (7.73)	27.92 (7.07)	28.50 (7.61)	27.32 (7.81)	0.32	0.811
Education (years)	12.76 (2.50)	11.74 (2.55)	12.50 (2.39)	12.64 (2.67)	2.44	0.064
Sex (male/female)	27/30	35/42	25/23	55/60	0.53^a^	0.913
Age of onset	21.48 (5.50)	22.24 (5.53)	22.85 (5.94)		0.71^b^	0.495
Duration of OCD (months)	75.95 (45.69)	65.83 (46.35)	63.60 (36.73)		1.41^b^	0.246
Total Y-BOCS	28.14 (5.61)	26.25 (4.37)	10.90 (2.34)		241.13^b^	<0.001*
Y-BOCS obsessions	14.47 (4.17)	14.61 (4.05)	5.12 (1.68)		119.45^b^	<0.001*
Y-BOCS compulsions	13.67 (4.17)	11.64 (4.04)	5.77 (1.95)		65.01^b^	<0.001*
HARS	10.49 (2.05)	10.26 (1.58)	5.48 (1.71)		134.49^b^	<0.001*
HDRS	6.04 (1.07)	5.43 (1.43)	2.94 (1.14)		91.02^b^	<0.001*

**Notes:** Abbreviations: nmOCD, non-medicated obsessive-compulsive disorder; mOCD, medicated obsessive-compulsive disorder; rOCD, remitted obsessive-compulsive disorder; HC, healthy controls; Y-BOCS, Yale-Brown Obsessive-Compulsive Scale; HARS, Hamilton Anxiety Rating Scale; HDRS, Hamilton Depression Rating Scale.

^a^χ^2^, df = 3.

^b^The comparison between the nmOCD, mOCD and rOCD groups.

*p < 0.001.

**Table 2:** Results of the neuropsychological tasks [M(S.D.)].

	nmOCD (n = 57)	mOCD (n = 77)	rOCD (n = 48)	HC (n = 115)	*F*	*p*	Effect size^a^
DST							
DST forward	9.11 (1.75)	9.09 (1.51)	9.75 (1.50)	9.43 (1.52)	2.30	0.077	0.20/0.22/0.21
DST backward	6.37 (1.54)	6.13 (1.20)	6.15 (1.13)	6.24 (1.37)	0.42	0.714	0.09/0.09/0.07
TMT							
TMT A(s)	35.75 (5.98)	34.67 (6.47)	34.20 (5.22)	35.18 (6.52)	0.65	0.584	0.09/0.08/0.17
TMT B(s)	73.87 (8.10)	70.42 (9.86)	68.82 (9.18)	71.35 (9.36)	2.85	0.038*	0.29/0.10/0.27
TMTA-TMTB(s)	38.12 (8.21)	35.75 (10.41)	34.63 (9.44)	36.17 (9.98)	1.23	0.301	0.21/0.04/0.16
WCST							
Total errors	48.42 (24.94)	47.49 (22.90)	43.40 (18.55)	50.83 (23.55)	1.23	0.299	0.10/0.14/0.35
Perseverative response	57.96 (30.56)	55.58 (28.44)	49.42 (23.99)	60.55 (29.61)	1.79	0.149	0.09/0.17/0.41
Perseverative errors	31.82 (21.27)	32.58 (21.68)	26.60 (21.84)	33.49 (19.94)	1.28	0.282	0.08/0.04/0.33

**Notes:** Abbreviations: nmOCD, non-medicated obsessive-compulsive disorder; mOCD, medicated obsessive-compulsive disorder; rOCD, remitted obsessive-compulsive disorder; HC, healthy controls; DST, Digit Span Test; TMT, Trail Making Test; WCST, Wisconsin Card Sorting Test.

^a^Effect size:small effect, ≤0.30; medium effect, 0.31–0.50; large effect, >0.50. The first number is the result of the comparison between the nmOCD and HC groups. The second number is the result of the comparison between the mOCD and HC groups. The third number is the result of the comparison between the rOCD and HC groups.

*p < 0.05.

**Table 3:** Decision making performances of the four groups [M(S.D.)].

	nmOCD (n = 57)	mOCD (n = 77)	rOCD (n = 48)	HC (n = 115)	*F*	*p*	Effect size^a^
IGT							
Block1	–2.88 (4.50)	–2.44 (5.19)	–2.29 (3.98)	–2.86 (4.00)	0.28	0.827	0.01/0.09/0.14
Block2	–1.72 (6.20)	–0.23 (6.08)	–0.04 (5.19)	–0.38 (5.53)	1.02	0.385	0.23/0.03/0.06
Block3	–0.84 (4.87)	–1.45 (4.60)	–0.62 (5.49)	0.99 (4.90)	4.35	0.005*	0.37/0.51/0.31
Block4	–0.96 (4.63)	–1.58 (5.96)	–1.92 (3.38)	2.37 (5.04)	14.21	< 0.001**	0.69/0.72/1.00
Block5	0.28 (4.59)	–0.75 (5.07)	–2.42 (3.52)	3.23 (6.81)	14.95	< 0.001**	0.51/0.66/1.04
Netscore	–6.12 (11.54)	–6.47 (14.09)	–7.29 (9.91)	3.35 (12.64)	15.03	< 0.001**	0.78/0.73/0.94
GDT							
One number	1.25 (2.17)	1.78 (3.12)	2.27 (3.71)	1.55 (2.63)	1.21	0.306	0.12/0.08/0.22
Two numbers	5.26 (3.54)	5.00 (3.93)	4.48 (3.24)	5.03 (4.14)	0.38	0.767	0.06/0.01/0.15
Three numbers	6.72 (3.01)	5.99 (3.62)	6.31 (3.45)	6.16 (3.88)	0.49	0.687	0.16/0.05/0.04
Four numbers	4.77 (4.01)	5.23 (4.43)	4.94 (3.92)	5.27 (4.69)	0.21	0.890	0.11/0.01/0.08
Netscore	4.98 (8.95)	4.44 (10.74)	4.50 (10.29)	4.85 (10.50)	0.05	0.987	0.01/0.04/0.03
Use of negative feedback^b^(%)	57.47 (40.71)	54.16 (36.16)	54.07 (38.06)	56.20 (38.73)	0.10	0.960	0.03/0.05/0.06
Use of positive feedback^c^(%)	61.06 (33.33)	63.15 (32.74)	59.01 (36.89)	64.29(33.46)	0.31	0.820	0.10/0.03/0.15

Notes: Abbreviations: nmOCD, non-medicated obsessive-compulsive disorder; mOCD, medicated obsessive-compulsive disorder; rOCD, remitted obsessive-compulsive disorder; HC, healthy controls; IGT, Iowa Gambling Task; GDT, Game of Dice Task.

^a^Effect size:small effect, ≤0.30; medium effect, 0.31–0.50; large effect, >0.50. The first number is the result of the comparison between the nmOCD and HC groups. The second number is the result of the comparison between the mOCD and HC groups. The third number is the result of the comparison between the rOCD and HC groups.

*p < 0.01, **p < 0.001.

^b^Sam*p*le size of the four groups (nmOCD: n = 53; mOCD: n = 61; rOCD: n = 40; HC: n = 99).

^c^Sample size of the four groups (nmOCD: n = 57; mOCD: n = 76; rOCD: n = 45; HC: n = 111).
